# Hydroxymethylated Phyllobilins: A Puzzling New Feature of the Dioxobilin Branch of Chlorophyll Breakdown

**DOI:** 10.1002/chem.201303398

**Published:** 2013-12-02

**Authors:** Iris Süssenbacher, Bastien Christ, Stefan Hörtensteiner, Bernhard Kräutler

**Affiliations:** [a] Institute of Organic Chemistry and Center for Molecular Biosciences, University of InnsbruckInnrain 80/82, 6020 Innsbruck (Austria); [b] Institute of Plant Biology, University of ZürichZollikerstrasse 107, 8008 Zürich (Switzerland)

**Keywords:** *Arabidopsis thaliana*, bilin, chlorophyll, cytochrome P450, phyllobilin

## Abstract

Colorless nonfluorescent chlorophyll (Chl) catabolites (NCCs) are formyloxobilin-type phyllobilins, which are considered the typical products of Chl breakdown in senescent leaves. However, in degreened leaves of some plants, dioxobilin-type Chl catabolites (DCCs) predominate, which lack the formyl group of the NCCs, and which arise from Chl catabolites by oxidative removal of the formyl group by a P450 enzyme. Here a structural investigation of the DCCs in the methylesterase16 mutant of *Arabidopsis thaliana* is reported. Eight new DCCs were identified and characterized structurally. Strikingly, three of these DCCs carry stereospecifically added hydroxymethyl groups, and represent bilin-type linear tetrapyrroles with an unprecedented modification. Indeed, DCCs show a remarkable structural parallel, otherwise, to the bilins from heme breakdown.

Chlorophyll breakdown is a visual sign of leaf senescence.[1–3] It is also an abundant source of linear chlorophyll-derived tetrapyrroles,[4] recently designated as phyllobilins,[5] structural relatives of the bilins from heme breakdown.[6] In degreened leaves of a variety of plants a linear path of chlorophyll breakdown appeared to be established, a few years ago, by which chlorophylls were degraded to formyloxobilin-type chlorophyll catabolites (CCs), and which eventually resulted in ubiquitous, (colorless) nonfluorescent CCs (NCCs) as main final products (Figure 1).[4,7,8] A key step in the formation of the phyllobilins is a characteristic oxygenolytic cleavage of the porphyrinoid macroring of the chlorophylls by pheophorbide a oxygenase (PaO), by which the *meso* carbon of the macrocycle is converted into a formyl group.[8,9] Indeed, to date, in higher plants no significant deviation from the common PaO/phyllobilin pathway[5] in the early phase of chlorophyll breakdown is known, which occurs in the chloroplast, and which furnishes ‘primary’ fluorescent CCs (*p*FCCs) as fleetingly existent intermediates (Figure 1).[5,7,10]

**Figure 1:**
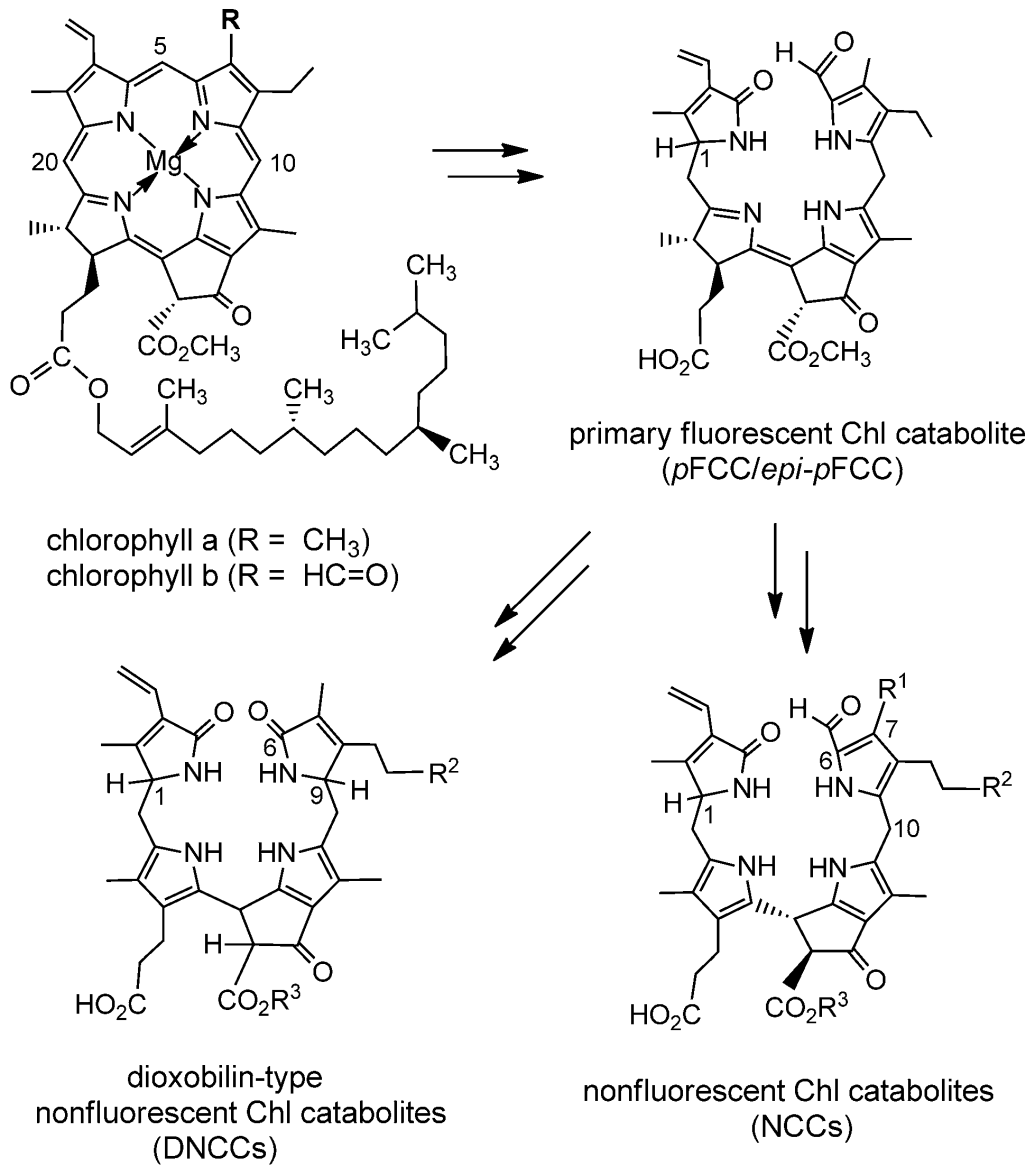
Abridged structural outline of chlorophyll breakdown in higher plants. Catabolite structures represent formyloxobilin-type catabolites, such as *p*FCC (*epi-p*FCC) and NCCs, and DNCCs. Atom numbering follows the convention with chlorophylls.[37]

In the ‘later’ steps of chlorophyll breakdown, which take place in the cytosol or in the vacuole, deviations from the established route from FCCs to NCCs have been observed more recently.[1,5] Thus, in senescent leaves of some tropical evergreens,[11] as well as in leaves[12] and peels of bananas[13,14] the striking accumulation of persistent hypermodified FCCs was observed. Also, in senescent leaves of Norway maple a dioxobilin-type NCC (DNCC) was found to accumulate, in which the formyl group was absent (Figure 1).[15] In some other senescent leaves, dioxobilin-type CCs (DCCs) were also found as major products of chlorophyll breakdown.[5,16,17] Indeed, in *Arabidopsis thaliana* colorless DCCs were detected recently as the dominant products of chlorophyll breakdown,[18] outweighing the earlier described NCCs from this plant.[19,20] The *Arabidopsis* enzyme that achieved the crucial deformylation reaction was identified as CYP89A9, a cytochrome P450 enzyme.[18] In in vitro experiments, CYP89A9 was shown to effectively catalyze the oxidative deformylation of the *p*FCC to the corresponding dioxobilin-type FCC (DFCC) providing a biochemical foundation for the formation of DCCs in *A. thaliana* leaves.[18]

Furthermore, in senescent leaves of *Arabidopsis methylesterase16* (MES16) was identified as the enzyme that hydrolyzes the methyl ester group of FCCs. Whereas a free acid group was present in the dominating CCs in the wild type,[19] the CCs in the *Arabidopsis mes16* mutant retained the methyl ester function of the chlorophylls.[21] Interestingly, a natural *mes16* mutant exists as the *Arabidopsis* Landsberg erecta (Ler) ecotype, which also lacks an obvious phenotype associated with the loss of activity of MES16.[21]

Here, we describe the analysis of fresh extracts of senescent leaves of the *Arabidopsis mes16* mutant.[21] Lack of the methylesterase MES16 in the mutant resulted in a less complex mixture of the CCs, simplifying analysis.[21] Indeed, as *p*FCC was the preferred substrate of the enzyme-catalyzed deformylation by CYP89A9,[18] the absence of MES16 was expected to have little effect on the crucial oxidative deformylation process. The study of a fresh extract of senescent leaves of the *mes16* mutant, reported here, revealed several novel colorless DCCs as the actual major chlorophyll breakdown products in this plant (besides the known FCCs and NCCs[21] as minor components), and allowed their structural characterization. Among these catabolites, a fluorescent DCC and several nonfluorescent DCCs were first characterized structurally. The structures of the fluorescent and of two of the nonfluorescent DCCs reflect puzzling (formal) hydroxymethylations, which are unprecedented among the known natural linear tetrapyrroles.

HPLC analysis of a fresh extract of senescent leaves (from 4 day dark incubations; Figure S1 in the Supporting Information) of the *Arabidopsis mes16* mutant revealed a variety of CCs (Figure 2). Several of these catabolites showed the typical absorbance at around 315 nm of formyloxobilin-type CCs, among them an FCC at *t*_R_=36.4 min ([*M*+H]^+^ ion at *m*/*z* 807.0), previously named *mes16*-FCC-1 (**1**), as well as an NCC at *t*_R_=39.7 min (*m*/*z* [*M*+H]^+^ 807.1), known as *mes16*-NCC-1 (**2**).[21] However, four major and three minor fractions had UV absorption bands near 237 and 274 nm, but none near 315 nm (for details see the Supporting Information). They were provisionally classified as nonfluorescent DCCs.[18] In addition, a fluorescent fraction was detected that exhibited only two absorption maxima near 237 nm and near 360 nm, and which was thus classified as a fluorescent DCC (Figure 2 and Figure 3). The newly characterized catabolites were named based on their source, their structure type and their retention times (analytical HPLC), for example, as *At-mes16*-DNCC-38 (**3**).

**Figure 2:**
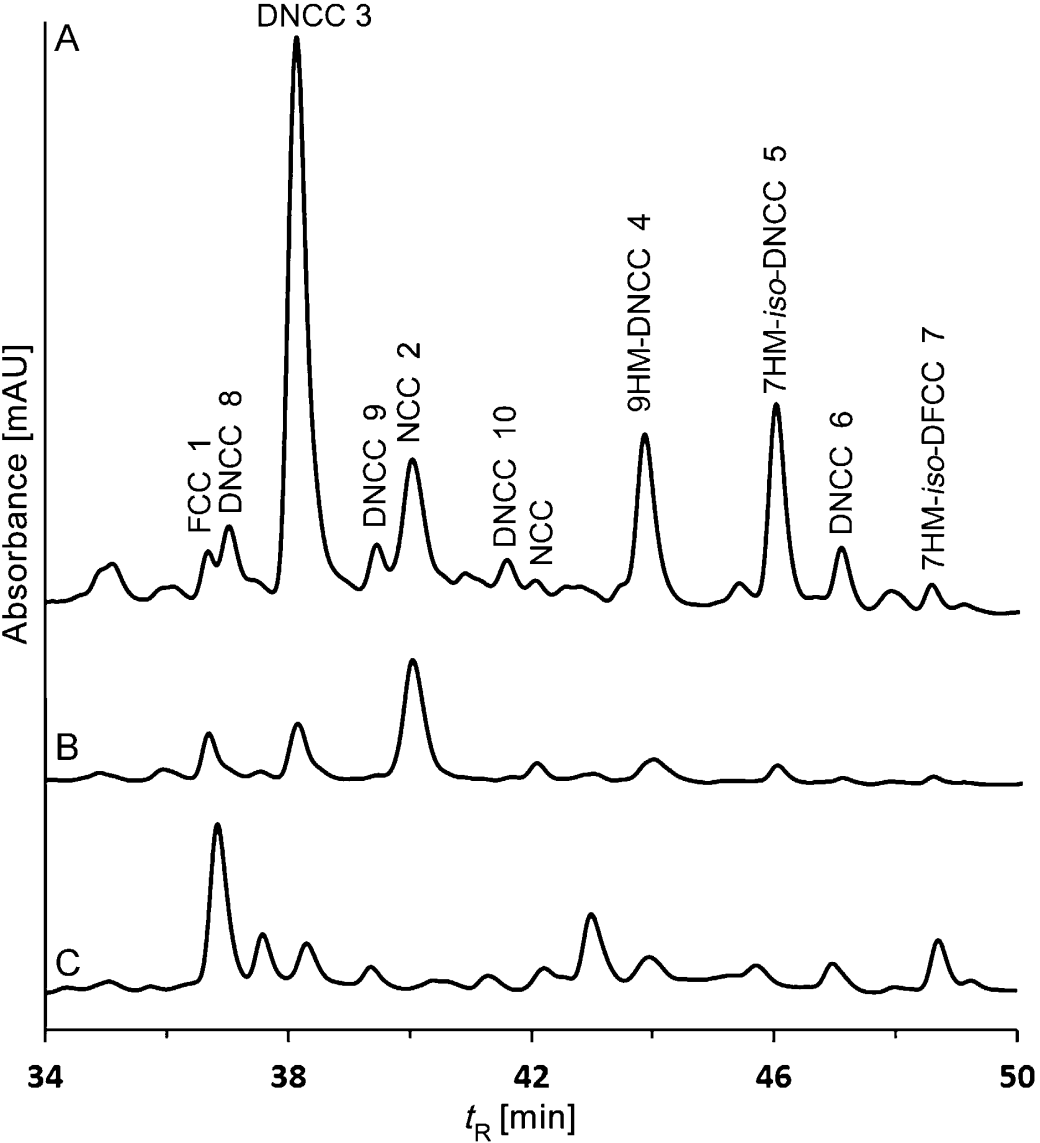
HPLC analysis of an extract of senescent leaves of *Arabidopsis mes16* with detection of absorbance at 254 nm (trace A) or 320 nm (trace B), and of luminescence at 450 nm (excitation at 350 nm, trace C). DNCCs give rise to a strong signal at 254 nm and a weak one at 320 nm (see Figure 3 for UV spectra).

**Figure 3:**
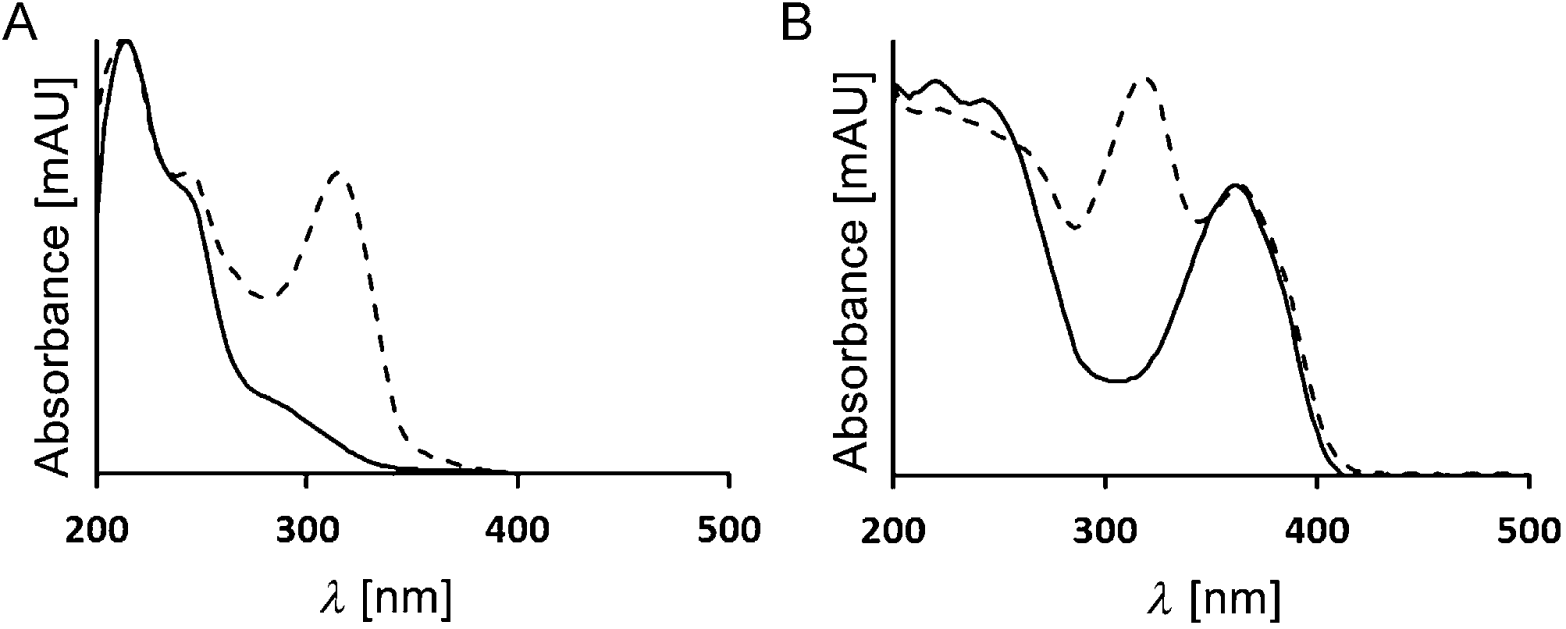
UV spectra of selected chlorophyll catabolites analyzed in senescent leaves of *Arabidopsis mes16*. Spectra of DCCs are represented by full lines, those of corresponding formyloxobilin-type catabolites by broken lines. A) Spectra of the DNCC 3 and of the NCC 2; B) spectra of the *iso*-DFCC 7 and of the FCC 1.

The major nonfluorescent DCC fractions with retention times (*t*_R_) of 38.1, 43.6, 45.7 and 46.8 min had UV spectra (Figure S2 in the Supporting Information) similar to those of structurally characterized DNCCs.[5,15–17] Their CD spectra were also similar to each other (see the Supporting Information) and had the same characteristics as those of several DCCs from senescent leaves of wild-type *Arabidopsis.*[18] The molecular formula (C_34_H_40_N_4_O_8_) of the polar catabolite at *t*_R_=38.1 min and designated here as *At-mes16*-DNCC-38 (**3**), was derived from its ESI mass spectrum ([*M*+H]^+^ ion at *m*/*z* 633.1). Two slightly less polar catabolites, *At-mes16*-9HM-DNCC-44 (**4**, *t*_R_=43.6 min) and *At-mes16*-7HM-*iso-*DNCC-46 (**5**, *t*_R_=45.7 min) were identified as isomers, as their mass spectra exhibited each an [*M*+H]^+^ ion at *m*/*z* 647.0, indicating a common molecular formula of C_35_H_42_N_4_O_8_. The molecular formula for the least polar nonfluorescent DCC, named *At*-*mes16*-DNCC-47 (**6**), was deduced as C_34_H_40_N_4_O_7_ from observation of the [*M*+H]^+^ ion at *m*/*z* 617.1. A fluorescent fraction at *t*_R_=48.2 min, and designated here as *At-mes16*-7HM-*iso-*DFCC (**7**), also exhibited a pseudo-molecular ion [*M*+H]^+^ at *m*/*z* 647.2 (corresponding to a molecular formula of C_35_H_42_N_4_O_8_ and identifying **7** as an isomer of **4** and of **5**).

^1^H NMR spectra of all catabolites investigated here, showed a singlet for a methyl ester group (consistent with the lack of the methylesterase MES16 in the *mes16* mutant),[21] and the typical signal pattern for a vinyl group, but a formyl hydrogen signal was absent. The molecular constitutions of *At-mes16*-DNCC-38 (**3**), *At-mes16*-9HM-DNCC-44 (**4**), *At-mes16*-7HM-*iso-*DNCC-46 (**5**), *At-mes16*-DNCC-47 (**6**) and of the fluorescent catabolite *At-mes16*-7HM-*iso-*DFCC (**7**), were deduced from homo- and heteronuclear 2D NMR spectra in CD_3_OD (^1^H,^1^H ROESY, ^1^H,^1^H COSY, ^1^H,^13^C HSQC and ^1^H,^13^C HMBC; Figure 4).

**Figure 4:**
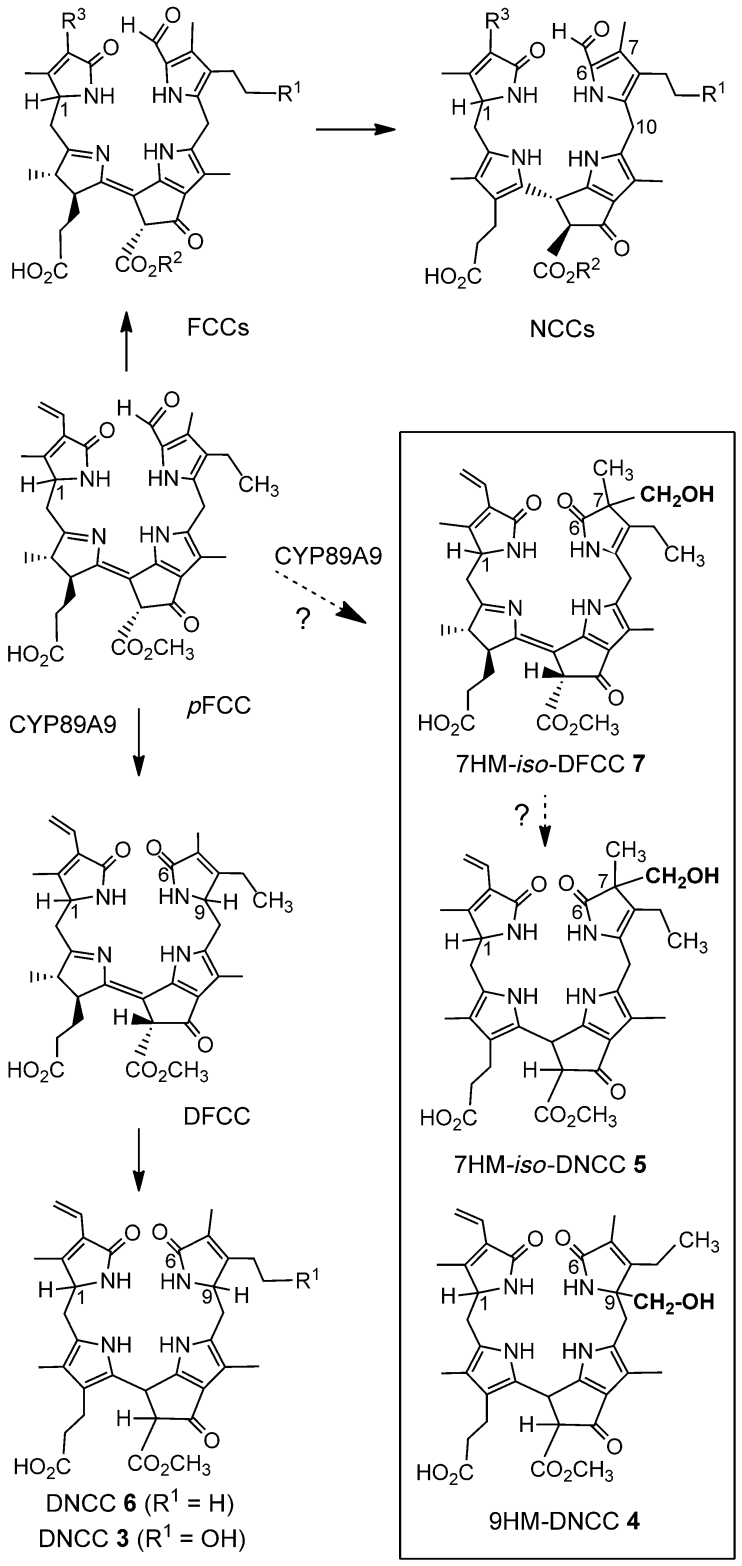
Steps downstream of *p*FCC of chlorophyll breakdown in leaves of the *Arabidopsis mes16* mutant. They lead either to formyloxobilin-type CCs or to dioxobilin-type CCs (represented in the Figure by structures in the upper or lower part, respectively). Structures of hydroxymethylated CCs 4, 5 and 7 of the *mes16* mutant and of their newly introduced hydroxymethyl groups are highlighted.

The ^1^H NMR spectrum of *At*-*mes16*-DNCC-38 (**3**) showed signals of 33 H atoms, that is, of all carbon-bound H atoms except for the one at the exchange labile 13^2^-position. A double doublet (dd) at *δ*=4.09 ppm and a triplet at *δ*=4.35 ppm indicated H atoms at the positions C-1 and C-9, typical for DNCCs.[15,16] Analysis of 2D NMR spectra (Figure S3 in the Supporting Information) revealed the constitution of *At*-*mes16*-DNCC-38 (**3**). It is a new natural DNCC (Figure 4). It is also the methyl ester of the major and most polar DCC from wild-type *Arabidopsis.*[18]

Signals of 35 of the 42 H atoms were detected in the 600 MHz ^1^H NMR spectrum of *At-mes16*-9HM-DNCC-44 (**4**). Among them was the spin system characteristic of a pyrrole-bound ethyl group. However, a signal near *δ*=4.3 ppm (typical of a DNCC, such as **3**) was absent. Instead, an AB system at *δ*=3.66/3.69 ppm was assigned to the CH_2_ moiety of a hydroxymethyl group. From analysis of the 2D NMR spectra, the hydroxymethyl group could be located at the C-9 position, and the ethyl group at C-8 (Figure S4 in the Supporting Information). The constitution of **4** could thus be elucidated: it had the same chromophore as **3**, but differed by the groups attached at the C-8 and C-9 positions. The nonfluorescent DCC **4** was thus indicated to have the unprecedented structure of a 9-hydroxymethyl-DNCC (9HM-DNCC), as shown in Figure 4.

In the ^1^H NMR spectrum of *At-mes16*-7HM-*iso-*DNCC-46 (**5**), signals of an ethyl group were again seen among the 35 H atoms observed, but that of an H atom at position C-9 (typical for a DNCC) was not. A dd at *δ*=4.10 ppm was confirmed to be due to an H atom at C-1. An AB system at *δ*=3.60/3.63 ppm (of a hydroxymethyl group) showed long-range correlations with the singlet at *δ*=1.06 ppm of the C-7 methyl group. Analysis of 2D NMR spectra (Figure S5 in the Supporting Information) revealed the structure of **5** as an isomer of 9HM-DNCC **4**, in which the positions of the hydroxymethyl group and of the remaining double bond at ring B were interchanged. Thus, **5** is a 7-hydroxymethyl-*iso*-DNCC (7HM-*iso-*DNCC, Figure 4). It is the first identified natural nonfluorescent *iso*-dioxobilin.

The chemical constitution of the DNCC **6** was likewise deduced from its NMR spectroscopy data (Figure S6 in the Supporting Information). It is the nonfluorescent isomer of the DFCC, which is generated from *p*FCC by deformylation by the cytochrome P450 enzyme CYP89A9[18] (constitutional formulas are shown in Figure 4).

The ^1^H NMR spectrum of the fluorescent DCC **7** showed signals of 37 carbon-bound H atoms. Assignments made from 2D NMR spectra (Figure S7 in the Supporting Information) were consistent with the structure of an FCC, except for the ring B moiety, in which, for example, a formyl group was lacking. However, an AB system at *δ*=3.62/3.64 ppm again indicated the diastereotopic protons of a methylene group, whereas the signal pattern of an ethyl side chain was also present. From correlations in 2D NMR spectra a hydroxymethyl group was indicated at C-7, next to a methyl group, and the ethyl group was located at C-8. Consistent with this, a double bond was indicated between C-8 and C-9. Thus, **7** was revealed to be a 7-hydroxymethyl-*iso*-DFCC (7HM-*iso-*DFCC, Figure 4) and rings B of **7** and of the 7HM-*iso-*DNCC **5** have the same structure. The catabolite **7** is a novel type of a fluorescent chlorophyll catabolite and an isomer (and, presumably, also direct precursor) of the nonfluorescent 7HM-*iso-*DNCC **5**.

Three further less abundant DNCCs (*At*-*mes16*-DNCC-37 (**8**), *At*-*mes16*-DNCC-40 (**9**), *At*-*mes16*-DNCC-42 (**10**)) were tentatively identified as epimers of the DNCC **3**, based on their UV and mass spectral properties (see the Supporting Information).

Eight DCCs could be characterized in the *Arabidopsis mes16* mutant. Five of them were classified as DNCCs: the DNCCs **3** and **6** were characterized structurally and three DNCCs (**8**–**10**) were provisionally identified as epimers of the DNCC **3**. The existence of these DNCCs suggests the intermediacy of corresponding DFCCs, from which DNCCs may arise by a stereoselective isomerization.[8,15,18] Presumably, the corresponding, but elusive, DFCCs are the result of an oxidative (CYP89A9 induced) deformylation of *p*FCC, followed (in the case of **3**) by enzyme-catalyzed hydroxylation at C-8^2^. Interestingly, the in vitro experiment with *p*FCC and CYP89A9 furnished two C-9 epimeric DFCCs,[18] suggesting a stereo-unselective protonation outside of this enzyme. This finding would be compatible with (hydrolytic) loss of the hypothetical C1 fragment occurring after release of the putative formate ester intermediate I, which could be directly generated by the monooxygenase (Figure 5). A stereo-unselective C-9 protonation of a likely (chemical?) deformylation intermediate, an α-hydroxypyrrole, was also inferred from the existence of two epimeric DNCCs (earlier called UCCs) in extracts of senescent barley leaves.[16]

**Figure 5:**
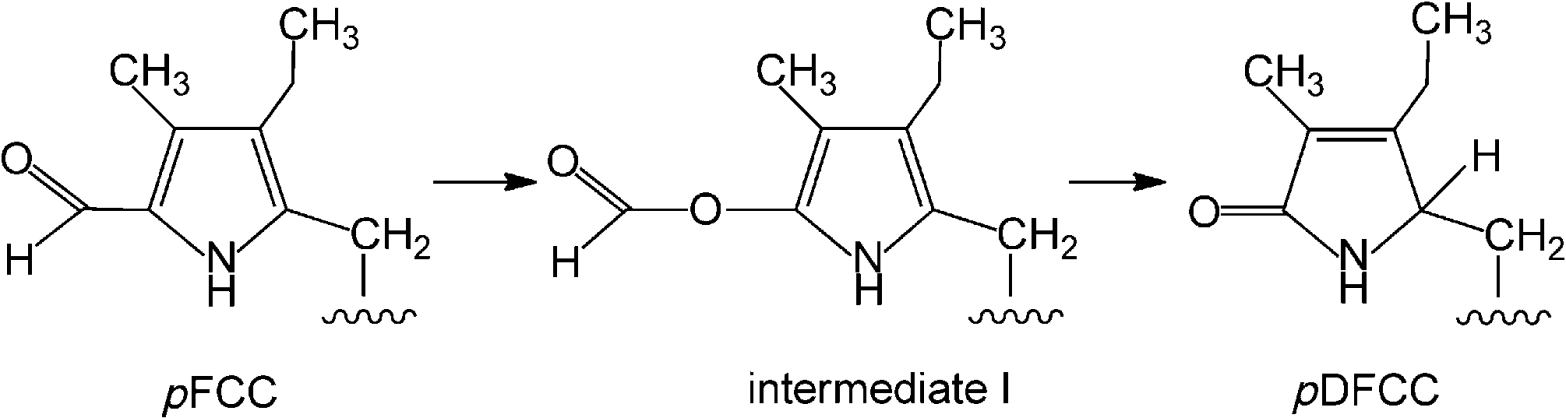
Possible steps of the oxidative deformylation at ring B of *p*FCC induced by the P450 enzyme CYP89A9 in *A. thaliana*.

Strikingly, the three further DCCs (9HM-DNCC (**4**), 7HM-*iso-*DNCC (**5**) and 7HM-*iso-*DFCC (**7**)) indicate stereoselective hydroxymethylation reactions, which neither have precedence in any of the previously characterized CCs (such as, for example, FCCs, NCCs) nor in heme-derived bilins.[6] Apparently, the remarkable (formal) hydroxymethylation reactions are directly associated with the appearance of DCCs. The structure of *At-mes16*-9HM-DNCC-44 (**4**) suggests a stereoselective hydroxymethylation at C-9, instead of the protonation seen in the DNCCs **3** and **6**. Indeed, the three DNCCs, **3**, **4** and **6** have the same basic chromophore. In contrast, **5** (a 7HM-*iso-*DNCC) and its presumed precursor, 7HM-*iso-*DFCC (**7**), reveal an alternative hydroxymethylation site at C-7. Indeed, a likely precursor of **7**, which could arise from hydrolytic deformylation at C-6 would have the proper reactivity at both, the C-7 and the C-9 positions for introduction of a hydroxymethyl group. The corresponding isomeric DCCs protonated at C-7 have not been observed, and an equivalent heme-derived *iso*-bilin-type tetrapyrrole is also unknown.[6,22] The lack of such *iso*-bilins[6] may be the result of their (presumed) inferior thermodynamic stability, when compared to the C-9 protonated—and known—isomers.[22,23] In contrast, hydroxymethylation introduces a substituent at C-7 that may be less prone to removal and migration to C-9. Thus, the here discovered (formal) tetrapyrrole hydroxymethylations appear to fix the chromophore structures of *iso-*DFCC and *iso-*DNCC effectively. Interestingly, DNCC **4** and *iso*-DNCC **5**, as well as *iso-*DFCC **7**, are consistently not modified further at C-8^2^ of their ethyl side chain. Apparently, the hydroxymethylations already provide a hydroxyl group that comes up (in an unknown functional and biosynthetic context) for the ubiquitous OH group at C-8^2^ (or an attached polar moiety, for example, a *O*-Glc), which is typical of most known phyllobilins.[4,5,24,25]

In line with the earlier finding that deformylation by CYP89A9 occurs preferably with FCCs still carrying a methyl ester function (i.e., before hydrolysis by MES16),[18] the here revealed structural peculiarities of several DCCs of the *Arabidopsis mes16* mutant are not a consequence of the absence of MES16. Indeed, the major DCC in wild-type *Arabidopsis* was identified earlier as analogue of **3**,[18] and two further representatives of these CCs appear to be analogues of the hydroxymethylated tetrapyrroles **4** and **5** (tentative characterization from UV and mass spectra). Ongoing work is directed at determining the structures of the new DCCs from wild-type *Arabidopsis*.

The remarkably stereoselective hydroxymethylation reactions of chlorophyll catabolites, discovered here, have no precedence from the heme-derived bilins.[6] This difference between heme and chlorophyll breakdown may be due to the particular biological functions of the two types of catabolites. Furthermore, as the entire path of bilin formation from heme does not involve a deformylation, the suggested biosynthetic availability of a reactive C1 fragment in the course of chlorophyll breakdown to DCCs,[18] lacks an equivalent in heme degradation. The observed additions to the DCC skeletons by hydroxymethylations at carbon[26] are unprecedented and truly exceptional catabolic steps.

The crucial deformylation reaction itself, which leads to DCCs, still needs to be further investigated. Although general precedence for the removal of formyl (and acyl) groups by P450 enzymes exists, there is none for the P450-catalyzed oxidative loss of a formyl group from the α-position of a pyrrole unit.[27,28] A nucleophilic (hydro)peroxo-Fe^III^ intermediate of the P450 cycle has been inferred to induce oxidative (C=C) bond cleavages.[28,29] The crucial step would thus be an oxygen insertion into the previous (C=C) bond with formation of a formate ester, reminiscent of the Bayer–Villiger reaction[30] (Figure 5). Hydrolysis of this putative ester and removal of the presently unknown C1 fragment (possibly formic acid) is likely to take place without assistance by the P450 enzyme.[27,28] Thus, it may occur after dissociation of the direct oxidation product from the enzyme and the deduced protonation at C-9 may take place in the aqueous environment. This scenario could explain the lack of stereoselectivity observed in the in vitro experiment with CYP89A9.[18] Alternatively, the removal of a C1 fragment from the hypothetical oxygenation intermediate could be catalyzed by (a) separate enzyme(s), for which this fragment could eventually serve as C1 component in further metabolism.[31] On the other hand, free formic acid, if generated by hydrolysis of the hypothetical oxygenation intermediate, would be considered a target of further metabolic detoxification in the plant to carbon dioxide.[32]

The three novel hydroxymethylated DCCs were detected as single stereoisomers, suggesting an enzyme-catalyzed formation of their new (C=C) bond. Their hydroxymethyl groups could eventually be derived from that intriguing hypothetical C1 fragment. However, the DCC structures suggest the requirement of the (formal) reduction of the formyl unit at C-6 to the oxidation level of formaldehyde. Possibly, a folate-based enzyme and donor of a formaldehyde equivalent would achieve this.[26,31] The structural data further suggest the generation of DCCs to occur by metabolically linked enzymatic steps in *Arabidopsis*, subsequent to the CYP89A9-catalyzed oxygen insertion. Clearly, the structures of the three hydroxymethylated DCCs suggest processes in this branch of chlorophyll breakdown in *A. thaliana* that are apparently unprecedented. Further biochemical and mechanistic studies are called upon to gain insights into this puzzle.

The transition from the original formyloxobilin-type CCs (such as FCCs, NCCs) to DCCs gives access to a second important downstream branch of the CCs (Figure 4). DCCs emphasize the close structural similarity between phyllobilins from chlorophyll, and the physiologically important bilins from heme catabolism,[6,22,33–35] also strengthening considerations of physiological roles of phyllobilins.[5,13] In this regard, not only formyloxobilin-type CCs are of interest, such as the persistent *hm*FCCs[13] and the ubiquitous NCCs,[4,36] but the dioxobilin-type CCs, as well.[5,16]

## Experimental Section

### Chemicals

See the Supporting Information.

### Plant material

*Arabidopsis mes16* was grown, as described.[21] Leaves were degreened in the dark (Figure S1 in the Supporting Information) and harvested, as described in the Supporting Information.

### HPLC methods

Hewlett Packard (hp) series 1100 HPLC system, online degasser, Agilent quaternary pump, diode array and fluorescence detector. Analytical HPLC (Figure 3): injection loop 200 μL (Rheodyne valve); Phenomenex hyperclone column ODS 5 μm 250×4.6 mm i.d.; Phenomenex precolumn ODS 4×3 mm i.d.; flow-rate 0.5 mL min^−1^. Solvent A: MeOH, solvent B: 10 mm ammonium acetate (NH_4_OAc) buffer; solvent composition A/B: 0–5 min: 20/80; 5–55 min: 20/80 to 70/30. Retention time (*t*_R_) in min; preparative HPLC: see the Supporting Information.

### Extraction and isolation of chlorophyll catabolites

Leaves of the *Arabidopsis mes16* mutant were kept in the dark for 4 days. An extract from 90 g (wet weight) of yellow-greenish leaves was used for the isolation of chlorophyll catabolites. Five major CC fractions were further purified to provide analytical samples of the DCCs **3**–**7**, as described below (see the Supporting Information).

### Spectroscopic analysis of chlorophyll catabolites

**General**: UV/Vis: Hitachi U-3000 spectrophotometer, in MeOH; *λ*_max_ [nm] (*ε*_rel_). NMR: Bruker UltraShield 600 MHz Avance II*+*. ESI-MS:[12] Finnigan LCQ classic, ESI source, positive ion mode, spray voltage 4.25 kV, MeOH/H_2_O (10 mm NH_4_OAc) 1:1 (v/v), *m*/*z* (%). For additional ESI-MS data, for CD and NMR spectroscopy data, see the Supporting Information.

***At-mes16*****-DNCC-38 (3)**: *t*_R_=38.1 min; UV/Vis (*c*=3.2×10^−5^ m): *λ*_max_ (*ε*_rel_)=286 sh (0.17), 236 sh (1.00), 216 nm (1.44); ESI-MS: *m*/*z* (%): 635.1 (12), 634.1 (40), 633.1 (100, C_34_H_41_N_4_O_8_^+^, [*M*+H]^+^); 601.0 (21, [*M*−CH_4_O+H]^+^); 510.0 (18, [*M*−C_7_H_9_NO (ring A)+H^+^]).

***At-mes16*****-9HM-DNCC-44 (4)***: t*_R_=43.6 min; UV/Vis (*c*=3.8×10^−5^ m): *λ*_max_ (*ε*_rel_)=294 sh (0.12), 238 sh (1.00), 216 nm (1.50); ESI-MS: *m*/*z* (%): 685.3 (26, [*M*+K]^+^); 669.3 (66, [*M*+Na]^+^); 649.1 (13), 648.1 (38), 647.0 (100, C_35_H_43_N_4_O_8_^+^, [*M*+H]^+^); 615.1 (19, [*M*−CH_4_O+H]^+^); 524.0 (9, [*M*−C_7_H_9_NO+H]^+^).

***At-mes16*****-7HM-*iso*-DNCC-46 (5)**: *t*_R_=45.7 min; UV/Vis (*c*=3.8×10^−5^ m): *λ*_max_ (*ε*_rel_)=284 sh (0.32), 238 sh (1.00), 216 nm (1.30); ESI-MS: *m*/*z* (%): 649.1 (13), 648.1 (41), 647.0 (100, C_35_H_43_N_4_O_8_^+^, [*M*+H]^+^); 617.0 (20, [*M*−CH_2_O+H]^+^); 615.1 (12, [*M*−CH_4_O+H]^+^); 585.2 (14, [*M*−C_2_H_6_O_2_+H]^+^); 524.1 (5, [*M*−C_7_H_9_NO+H]^+^).

***At-mes16*****-DNCC-47 (6)**: *t*_R_=46.8 min; UV/Vis (*c*=3.2×10^−5^ m): *λ*_max_ (*ε*_rel_)=294 sh (0.23), 242 sh (1.00), 219 nm (1.36); ESI-MS: *m*/*z* (%): 619.1 (11), 618.1 (39), 617.2 (100, C_34_H_41_N_4_O_7_^+^, [*M*+H]^+^); 585.1 (26, [*M*−CH_4_O+H]^+^); 494.1 (11, [*M*−C_7_H_9_NO (ring A)+H]^+^).

***At-mes16*****–7HM-*iso*-DFCC (7)**: *t*_R_=48.2 min; UV/Vis (*c*=1.8×10^−5^ m): *λ*_max_ (*ε*_rel_)=358 (1.00), 244 (1.28), 222 nm (1.34); ESI-MS: *m*/*z* (% intensity, type of ion): 685.1 (22, [*M*+K]^+^); 669.2 (14, [*M*+Na]^+^); 649.1 (18), 648.2 (49), 647.2 (100, C_35_H_43_N_4_O_8_^+^, [*M*+H]^+^); 617.2 (22, [*M*−CH_2_O+H]^+^); 615.0 (17, [*M*−CH_4_O+H]^+^); 585.2 (12, [*M*−C_2_H_6_O_2_+H]^+^); 494.1 (10, [*M*−C_8_H_11_NO_2_+H]^+^); 492.13 (10, [*M*−C_8_H_13_NO_2_+H]^+^).
