# Supplementary material for: Hydroxymethylated Phyllobilins: A Puzzling New Feature of the Dioxobilin Branch of Chlorophyll Breakdown
Source: Chemistry. 2013 Dec 2;20(1):87–92. doi: 10.1002/chem.201303398 (PMC4497322; doi:10.1002/chem.201303398)
Supplement: Supplementary file 1 [file chem0020-0087-sd1.pdf]

CHEMISTRY
A EUROPEAN JOURNAL

Supporting Information

© Copyright Wiley-VCH Verlag GmbH & Co. KGaA, 69451 Weinheim, 2013

Hydroxymethylated Phyllobilins: A Puzzling New Feature of the Dioxobilin Branch of Chlorophyll Breakdown

Iris Süssenbacher,[a] Bastien Christ,[b] Stefan Hörtensteiner,[b] and Bernhard Kräutler*[a]

chem_201303398_sm_miscellaneous_information.pdf

Supporting Information

Materials and Methods

Chemicals

HPLC-grade methanol (MeOH) and n-hexane were from *VWR* (Leuven, Belgium) and *Acros Organics* (Geel, Belgium). Potassium dihydrogen phosphate puriss. p.a, potassium phosphate dibasic-anhydrous puriss. p.a and ammonium acetate were from *Fluka* (Buchs, Switzerland). Ultrapure water (18 $M\Omega cm^{-1}$) was from a *Millipore* apparatus, 5g and 1g Sep-Pak-C18 Cartridges from *Water Associates* (Milford, USA).

Plant Material

The *mes16-1* mutant of *Arabidopsis thaliana* [1] was grown on soil in a controlled growth chamber at 22°C and 60% relative humidity under short day conditions (8 h light/16 h dark) with fluorescent light of 60-120 µmol photons m^{-2} s^{-1}. For senescence induction, detached leaves from 8-week-old plants were incubated in darkness on wet filter paper for 4 d.

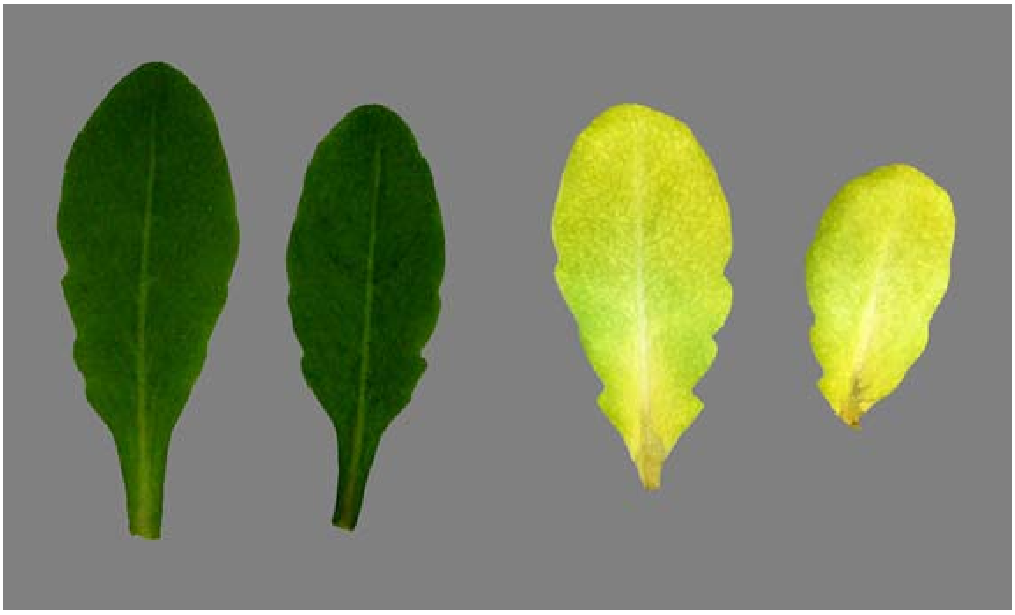

Figure S1: Pictures of green (left) and yellow senescent (right) *mes16-1* leaves collected after 4 d dark incubation.

Chromatographic Methods

<u>HPLC</u>. *Hewlett Packard* (*hp*) series 1100 HPLC-system, online degasser, *Agilent* quaternary pump, diode array detector (DAD) and fluorescence detector (FLD). *Analytical HPLC:* Injection loop 200 µL (*Rheodyne* valve); *Phenomenex hyperclone* ODS 5 µm 250 x 4.6 mm i.d. column (at room temperature) connected to *Phenomenex* ODS 4 x 3 mm i.d. pre-column

was used with a flow rate of 0.5 ml min^{-1}. Solvent A: MeOH, solvent B: 10 mM ammonium acetate puffer standard solvent composition A/B: 0 – 5 min: 20/80; 5 – 55 min: 20/80 to 70/30; 55 – 60 min: 70/30 to 100/0; 60 – 70 min: 100/0; 70 – 75 min: 100/0 to 20/80. Preparative HPLC: Injection loop 1.2 ml; *Phenomenex hyperclone* ODS 5 µm 250 x 21.2 mm id. column (at room temperature) protected with a *Phenomenex* ODS 10 x 5 mm pre-column was used with a flow rate of 5 ml min^{-1}. Data were collected and processed with *Agilent ChemStation*. Solvent A: MeOH, solvent B: 10 mM potassium phosphate buffer (pH 7); solvent composition A/B: 0 – 10 min: 20/80; 10 – 210 min: 20/80 to 55/45; 210 – 240 min: 55/45 to 63/37; 240 – 250 min: 63/37 to 100/0; 250 – 260 min: 100/0; 260 – 270 min: 100/0 to 20/80.

Extraction and Isolation of Chlorophyll Catabolites

90 g (wet weight) of frozen, powdered yellow-greenish leaves of the *Arabidopsis thaliana mes16* mutant (kept in darkness for 4 days) were mixed with sea sand and extracted with 30 ml MeOH. The obtained slurry was filtrated through a Buchner funnel and the extraction with 10 ml MeOH was repeated 8 times. The collected green extract was diluted with 60 ml potassium phosphate buffer (50 mM, pH 7) and washed two times with 120 ml n-hexane. The solution was diluted with 600 ml of potassium phosphate buffer (50mM, pH 7) and filtrated. The mixture was loaded on a Sep-Pak Vac 20cc (5g) C18 cartridge, washed with water (50 ml) and eluted with MeOH (15 ml). The solvent was removed under reduced pressure on a rotary evaporator. The crude product was dissolved in 4 ml MeOH/potassium phosphate buffer (50:50 *v*/*v*) and portioned into 4 fraction of about 1 ml, which were centrifuged for 5 min at 13 000 rpm. The clear brown solution was injected into the preparative HPLC system. The relevant raw fractions from 6 runs were collected and analyzed by HPLC. Raw catabolite fractions, *At-mes16*-DNCC-38 (**3**), *At-mes16*-9HM-DNCC-44 (**4**), *At-mes16*-7HM-*iso*-DNCC-46 (**5**), *At-mes16*-DNCC-47 (**6**) and *At-mes16*-7HM-*iso*-DFCC (**7**) were diluted with water, concentrated on a Sep-Pak classic C18 cartridge and re-purified by means of analytical HPLC. The collected fractions of each catabolite were diluted with 4 volumes of water, applied to a Sep-Pak classic C18 cartridge, washed with 20 ml H_2O and eluted with 5 ml MeOH. The solvents were removed in vacuum and analytically pure samples of *At-mes16*-DNCC-38 (**3**, 2.3 mg), *At-mes16*-9HM-DNCC-44 (**4**, 1.0 mg), *At-mes16*-7HM-*iso*-DNCC-46 (**5**, 1.0 mg), *At-mes16*-DNCC-47 (**6**, 0.7 mg) and *At-mes16*-7HM-*iso*-DFCC (**7**, 0.4 mg) were obtained.

Spectroscopic Analysis of Chlorophyll Catabolites

General. Ultraviolet/visible (UV/Vis): *Hitachi U-3000* spectrophotometer, in MeOH; λ_{max} [nm](ε_{rel}). *Circular dichroism (CD)*: *JASCO J715*, in MeOH; λ $_{min/max}$ [nm],($\Delta\varepsilon$). *Nuclear magnetic resonance* (*NMR*): *Bruker UltraShield 600 MHz Avance II+* or *Varian Unity Inova 500 MHz* spectrometers, ^{1}H-NMR (in CD_3OD, at 285K, δ ($C^{1}\mathit{H}D_2COD$) = 3.31 ppm [2]; s, d, dd, t, m = singlet, doublet, double doublet, triplet, multiplet, assignment from ^{1}H,^{1}H-COSY and ^{1}H,^{1}H-ROESY spectra); ^{13}C-NMR (in CD_3OD, at 285K, δ($^{13}\mathit{C}D_3OD$) = 49.0 ppm [2], assignment of signals from ^{1}H,^{13}C-HSQC and ^{1}H,^{13}C-HMBC spectra) [3,4]. Electrospray ionization mass spectra (ESI-MS) [5]: *Finnigan LCQ classic*, ESI-source, positive ion mode, spray voltage 4.25 kV, solvent MeOH/H_2O (10 mM NH_4OAc) 1:1 (*v/v*), m/z (% intensity, type of ion), signals of isotopomeric ions are listed only for quasi molecular ions ($[M+H]^{+}$). Atom numbering follows the convention of chlorophylls [6].

UV-spectral data of four main nonfluorescent DCC-fractions

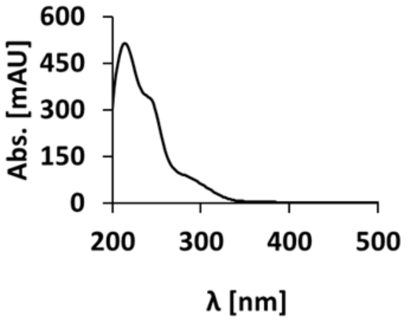

At-mes16-DNCC-38 (**3**)

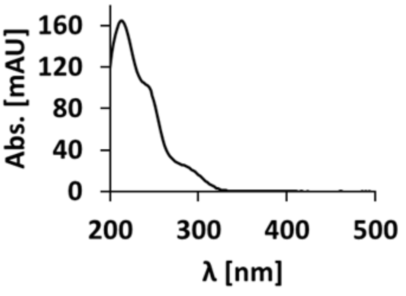

At-mes16-9HM-DNCC-44 (**4**)

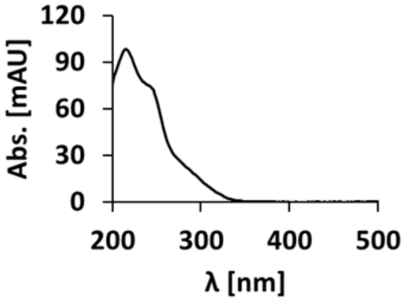

At-mes16-7HM-*iso*-DNCC-46 (**5**)

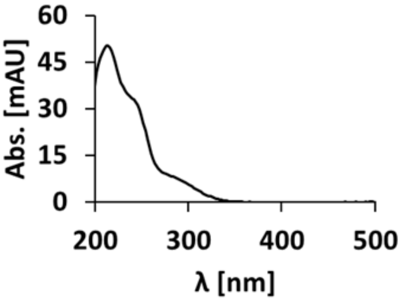

At-mes16-DNCC-47 (**6**)

Figure S2. Online UV/Vis spectra of main nonfluorescent DCCs (*At-mes16*-DNCC-38 (**3**), *At-mes16*-9HM-DNCC-44 (**4**), *At-mes16*-7HM-*iso*-DNCC-46 (**5**) and *At-mes16*-DNCC-47(**6**))

Spectral data of *At-mes16*-DNCC-38 (**3**), a $3^1,3^2$-didehydro-8^2-hydroxy-1,4,5,10,15,20,22,24-(9H,21H,23H)-octahydro-13^2-methoxycarbonyl-4,5-seco-5-nor-4,6-dioxo-phytoporphyrinate (see main text, Figure 4). UV/Vis (c = 3.2*10^{-5} M): λ_{max} (ε_{rel}) = 286 sh (0.17), 236 sh (1.00), 216 (1.44). CD (c = 3.2*10^{-5} M): $\lambda_{min/max}$ [nm] ($\Delta\varepsilon$) = 311 (3.7), 284 sh (-17.8), 252 sh (1.2), 228 (20.0), 205 (-22.9). NMR (see figure S3): 500 MHz ^{1}H-NMR: δ [ppm] = 1.77 (s, H_3C-7^1), 1.96 (s, H_3C-18^1), 1.99 (s, H_3C-2^1), 2.11 (s, H_3C-12^1), 2.36 (m, H_2C-17^2), 2.45 (dd, J = 14.5/9.1 Hz, H_AC-20), 2.50 (m, H_AC-8^1), 2.54 (dd, J = 8.8/14.7 Hz, H_AC-10), 2.64 (m, H_AC-17^1), 2.72 (m, H_BC-17^1), 2.79 (m, H_BC-8^1), 2.91 (dd, J = 14.5/4.8 Hz, H_BC-20), 3.08 (dd, J = 14.8/4.6 Hz, H_BC-10), 3.70 (m, H_2C-8^2), 3.75 (s, H_3C-13^5), 4.09 (dd, J = 9.1/4.8 Hz, HC-1), 4.35 (dd, J ~ 8.7/4.7 Hz, HC-9), 4.93 (s, HC-15), 5.32 (dd, J = 11.8/2.0 Hz, H_AC-3^2), 6.08 (dd, J = 17.8/2.0 Hz, H_B-3^2)), 6.44 (dd, J = 17.8/11.8 Hz, HC-3^1). 125 MHz ^{13}C-NMR: δ [ppm] = 8.0 (7^1), 8.9 (18^1), 9.5 (12^1), 12.1 (2^1), 21.2 (17^1), 30.0 (10), 30.2 (20), 31.0 (8^1), 36.7 (15), 38.4 (17^2), 52.3 (13^5), 60.5 (9), 60.8 (8^2), 61.3 (1), 67.5 (13^2), 112.5 (12), 114.9 (18), 118.7 (3^2), 120.3 (17), 123.9 (16), 124.2 (19), 125.6 (13), 126.9 (3^1), 128.3 (3), 130.5 (7), 134.4 (11), 155.6 (8), 156.5 (2), 160.9 (14), 171.4 (13^3), 174.2 (4), 176.2 (6), 180.0 (17^3). ESI-MS: m/z (%) = 671.2 (17, $[M+K]^+$); 655.2 (5, $[M+Na]^+$); 635.1 (12), 634.1 (40), 633.1 (100, $C_{34}H_{41}N_4O_8^+$, $[M+H]^+$); 601.0 (21, $[M-CH_4O+H]^+$); 510.0 (18, [M-C_7H_9NO (ring A)+H]$^+$); 478.1 (5, $[M-C_8H_{13}NO_2 + H]^+$).

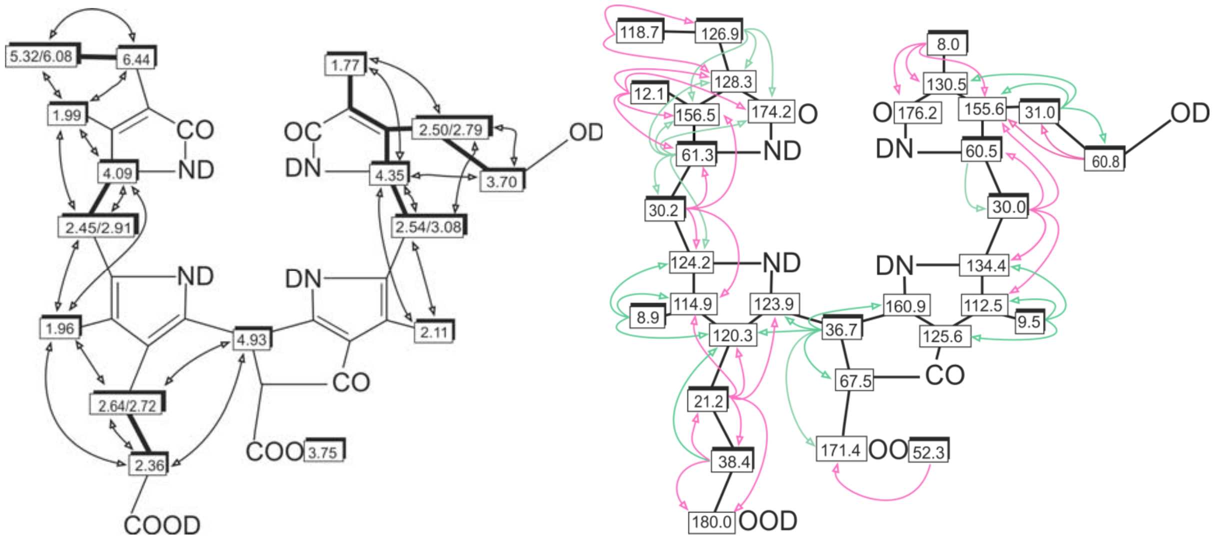

Figure S3. Graphical structural analysis of *At-mes16*-DNCC-38 (**3**). Left. ^{1}H-chemical shift assignments from ^{1}H,^{1}H-ROESY and COSY correlations (arrows or bold bonds, resp.). Right. ^{13}C chemical shift assignments from ^{1}H,^{13}C-HSQC correlations are shown by shaded boxes, and arrows indicate ^{1}H,^{13}C-HMBC long range correlations.

<u>Spectral data of *At-mes16*-9HM-DNCC-44 (**4**),</u> a 3^1,3^2-didehydro-9-hydroxymethyl-1,4,5,10,15,20,22,24-(9H,21H,23H)-octahydro-13^2-methoxycarbonyl-4,5-seco-5-nor-4,6-dioxo-phytoporphyrinate (see main text, Figure 4). UV/Vis (c = 3.8*10^{-5} M): λ_{max} (ε_{rel}) = 294 sh (0.12), 238 sh (1.00), 216 (1.50). CD (c = 3.8*10^{-5} M): $\lambda_{min/max}$ [nm] ($\Delta\varepsilon$) = 310 (4.7), 284 (-16.3), 252 sh (2.5), 225 (26.8), 207 (-37.4). NMR (see Figure S4) 600 MHz ^{1}H-NMR: δ [ppm] = 1.15 (t, J = 7.7 Hz, H_3C-8^2), 1.74 (s, H_3C-7^1), 1.97 (s, H_3C-18^1), 2.02 (s, H_3C-2^1)), 2.05 (s, H_3C-12^1), 2.38 (m, H_2C-17^2), 2.42 (m, H_AC-8^1), 2.45 (dd, J = 14.5/ 9.3 Hz, H_AC-20), 2.50 (m, H_BC-8^1), 2.65 (m, H_AC-17^1), 2.71 (m, H_BC-17^1), 2.80/3.06 (AB-system, J = 15.2 Hz, H_2C-10), 2.99 (dd, J= 14.5/4.6 Hz, H_BC-20), 3.66/3.69 (AB-system, J = 11.3 Hz, H_2C-9^1), 3.74 (s, H_3C-13^5), 4.13 (dd, J = 9.3/4.6 Hz, HC-1), 4.92 (s, HC-15), 5.33 (dd, J= 11.7/2.3 Hz, H_AC-3^2), 6.10 (dd, J = 17.7/2.3 Hz, H_BC-3^2), 6.45 (dd, J = 17.7/11.7 Hz, HC-3^1). 150 MHz ^{13}C-NMR: δ [ppm] = 8.3 (7^1), 9.2 (18^1), 9.6 (12^1), 12.4 (2^1), 12.6 (8^2), 20.0 (8^1), 21.2 (17^1), 30.2 (20), 30.4 (10), 37.1 (15), 38.3 (17^2), 52.6 (13^5), 61.7 (1), 64.6 (9^1), 67.6 (13^2), 70.2 (9), 113.6 (12), 115.0 (18), 118.8 (3^2), 120.3 (17), 123.8 (16), 124.2 (19), 125.4 (13), 126.8 (3^1), 128.4 (3), 130.1 (7), 132.8 (11), 156.6 (2), 160.6 (8), 160.9 (14), 171.4 (13^3), 174.6 (4), 176.0 (6), 179.6 (17^3). ESI-MS: m/z (%) = 685.27 (26, $[M+K]^+$); 669.27 (66, $[M+Na]^+$); 649.1 (13), 648.1 (38), 647.0 (100, $C_{35}H_{43}N_4O_8^+$, $[M+H]^+$); 615.1 (19, $[M-CH_4O+H]^+$); 524.0 (9, $[M-C_7H_9NO+H]^+$); 492.2 (5, $[M-C_8H_{13}NO_2+H]^+$); 460.13 (3, $[M-C_9H_{17}NO_3 + H]^+$); 369.20 (3, $[M-C_{14}H_{20}N_2O_3+H]^+$).

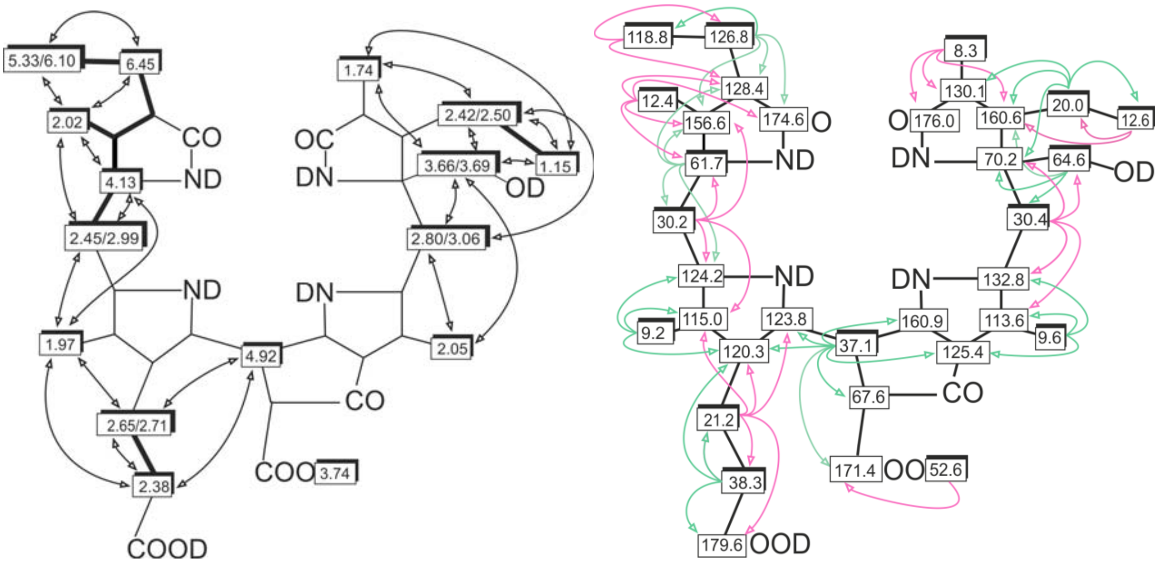

Figure S4. Graphical structural analysis of *At-mes16*-9HM-DNCC-44 (**4**). Left. ^{1}H-chemical shift assignments from ^{1}H,^{1}H-ROESY and COSY correlations (arrows or bold bonds, resp.). Right. ^{13}C chemical shift assignments from ^{1}H,^{13}C-HSQC correlations are shown by shaded boxes, and arrows indicate ^{1}H,^{13}C-HMBC long range correlations.

Spectral data of *At-mes16*-7HM-*iso*-DNCC-46 (**5**), $3^1,3^2$-didehydro-7-hydroxymethyl-1,4,5,10,15,20,22,24-(7H,21H,23H)-octahydro-13^2-methoxycarbonyl-4,5-seco-5-nor-4,6-dioxo-phytoporphyrinate (see main text, Figure 4). UV/Vis (c = 3.8*10^{-5} M): λ_{max} (ε_{rel}) = 284 sh (0.32), 238 sh (1.00), 216 (1.30). CD (c = 3.8*10^{-5} M): $\lambda_{min/max}$ [nm] ($\Delta\varepsilon$) = 312 (3.1), 284 (-12.9), 252 (5.5), 226 (18.9), 207 (-17.4). NMR (see Figure S5): 600 MHz ^{1}H-NMR: δ [ppm] = 1.06 (s, H_3C-7^1), 1.07 (t, J = 7.6 Hz, H_3C-8^2), 1.94 (s, H_3C-18^1), 1.96 (s, H_3C-2^1), 2.14 (s, H_3C-12^1), 2.20 (m, H_AC-8^1), 2.26 (m, H_BC-8^1), 2.33 (m, H_2C-17^2), 2.52 (dd, J = 14.6/8.6 Hz, H_AC-20), 2.63 (m, H_AC-17^1), 2.67 (m, H_BC-17^1), 2.85 (dd, J = 14.6/5.2 Hz, H_BC-20), 3.57/3.66 (AB-system, J = 17.2 Hz, H_2C-10), 3.60/3.63 (AB-system, J = 11.4 Hz, H_2C-$7^{1'}$), 3.74 (s, H_3C-13^5), 4.10 (dd, J = 8.6/5.2 Hz, HC-1), 4.86 (s, HC-15), 5.34 (dd, J = 11.7/2.2 Hz, H_AC-3^2), 6.11 (dd, J = 17.6/2.3 Hz, H_BC-3^2), 6.45 (dd, J = 17.7/11.6 Hz, HC-3^1). 150 MHz ^{13}C-NMR: δ [ppm] = 9.1 (12^1), 9.1 (18^1), 12.3 (2^1), 15.0 (8^2), 17.6 (8^1), 17.7 (7^1), 21.4 (17^1), 23.1 (10), 30.0 (20), 37.2 (15), 38.4 (17^2), 52.6 (13^5), 55.5 (7), 61.4 (1), 65.9 ($7^{1'}$), 67.5 (13^2), 111.9 (12), 115.0 (18), 118.8 (3^2), 119.9 (17), 122.9 (8), 123.8 (16), 123.9 (19), 125.6 (13), 126.7 (3^1), 128.2 (3), 132.5 (9), 132.7 (11), 156.5 (2), 160.2 (14), 171.3 (13^3), 174.3 (4), 179.4 (17^3), 184.5 (6). ESI-MS: m/z (%) = 685.1 (21, $[M+K]^+$); 669.1 (6, $[M+Na]^+$); 649.1 (13), 648.1 (41), 647.0 (100, $C_{35}H_{43}N_4O_8^+$, $[M+H]^+$); 617.0 (20, $[M-CH_2O+H]^+$); 615.1 (12, $[M-CH_4O+H]^+$); 585.2 (14, $[M-C_2H_6O_2+H]^+$); 524.1 (5, $[M-C_7H_9NO+H]^+$); 492.1 (5, $[M-C_8H_{13}NO_2+H]^+$).

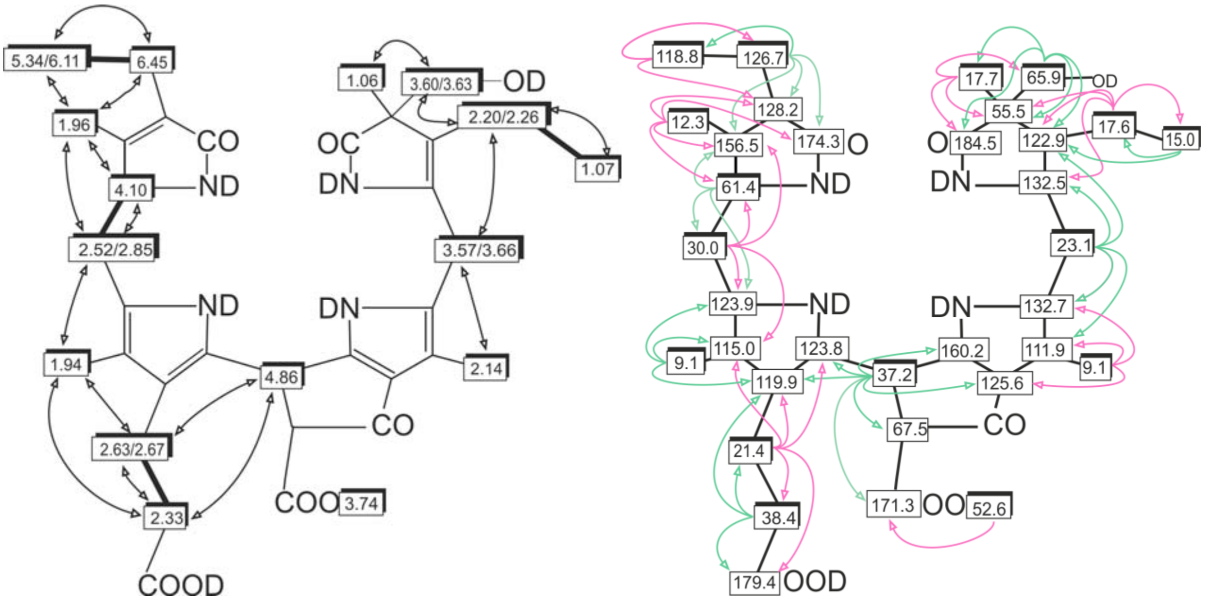

Figure S5. Graphical structural analysis of *At-mes16*-7HM-*iso*-DNCC-46 (**5**). Left. ^{1}H-chemical shift assignments from ^{1}H,^{1}H-ROESY and COSY correlations (arrows or bold bonds, resp.). Right. ^{13}C chemical shift assignments from ^{1}H,^{13}C-HSQC correlations are shown by shaded boxes, and arrows indicate ^{1}H,^{13}C-HMBC long range correlations.

Spectral data of *At-mes16*-DNCC-47 (**6**), $3^1,3^2$-didehydro-1,4,5,10,15,20,22,24-(9H, 21H,23H)-octahydro-13^2-methoxycarbonyl-4,5-seco-5-nor-4,6-dioxo-phytoporphyrinate (see main text, Figure 4). UV/Vis (c = 3.2*10^{-5} M): λ_{max} (ε_{rel}) = 294 sh (0.23), 242 sh (1.00), 219 (1.36). CD (c = 3.2*10^{-5} M): $\lambda_{min/max}$ [nm] ($\Delta\varepsilon$) = 310 (1.32), 284 (-5.00), 255 sh (0.85), 228 (5.93), 206 (-7.44). NMR (see figure S6): 600 MHz ^{1}H-NMR: δ [ppm] = 1.13 (t, J = 7.6 Hz, H_3C-8^2), 1.75 (s, H_3C-7^1), 1.96 (s, H_3C-18^1), 1.99 (s, H_3C-2^1), 2.11 (s, H_3C-12^1), 2.35 (m, H_AC-8^1), 2.36 (m, H_2C-17^2), 2.47 (dd, J = 14.5/9.2 Hz, H_AC-20), 2.51 (dd, J = 8.9/14.8 Hz, H_AC-10), 2.59 (m, H_BC-8^1), 2.66 (m, H_AC-17^1), 2.72 (m, H_BC-17^1), 2.90 (dd, J = 14.5/4.9 Hz, H_BC-20), 3.08 (dd, J = 14.8/4.3 Hz, H_BC-10)), 3.75 (s, H_3C-13^5), 4.10 (dd, J = 9.1/4.9 Hz, HC-1), 4.30 (dd, J = 8.9/4.3 Hz, HC-9), 4.93 (s, HC-15), 5.33 (dd, J = 11.7/2.3 Hz, H_AC-3^2), 6.08 (dd, J = 17.7/2.3 Hz, H_BC-3^2), 6.45 (dd, J = 17.8/11.6 Hz, HC-3^1). 150 MHz ^{13}C-NMR (only HSQC): δ [ppm] = 8.1 (7^1), 9.2 (18^1), 9.3 (12^1), 12.4 (2^1), 13.1 (8^2), 20.5 (8^1), 21.6 (17^1), 29.8 (10), 30.2 (20), 37.2 (15), 38.9 (17^2), 52.6 (13^5), 59.6 (9), 61.5 (1), 118.9 (3^2), 126.8 (3^1). ESI-MS: m/z (%) = 655.2(29, $[M+K]^+$); 639.2 (11, $[M+Na]^+$); 619.1 (11); 618.1 (39), 617.2 (100, $C_{34}H_{41}N_4O_7^+$, $[M+H]^+$); 585.1 (26, $[M-CH_4O+H]^+$); 494.1 (11, [M-C_7H_9NO (ring A)+H]$^+$); 462.1 (7, $[M-C_8H_{13}NO_2+H]^+$).

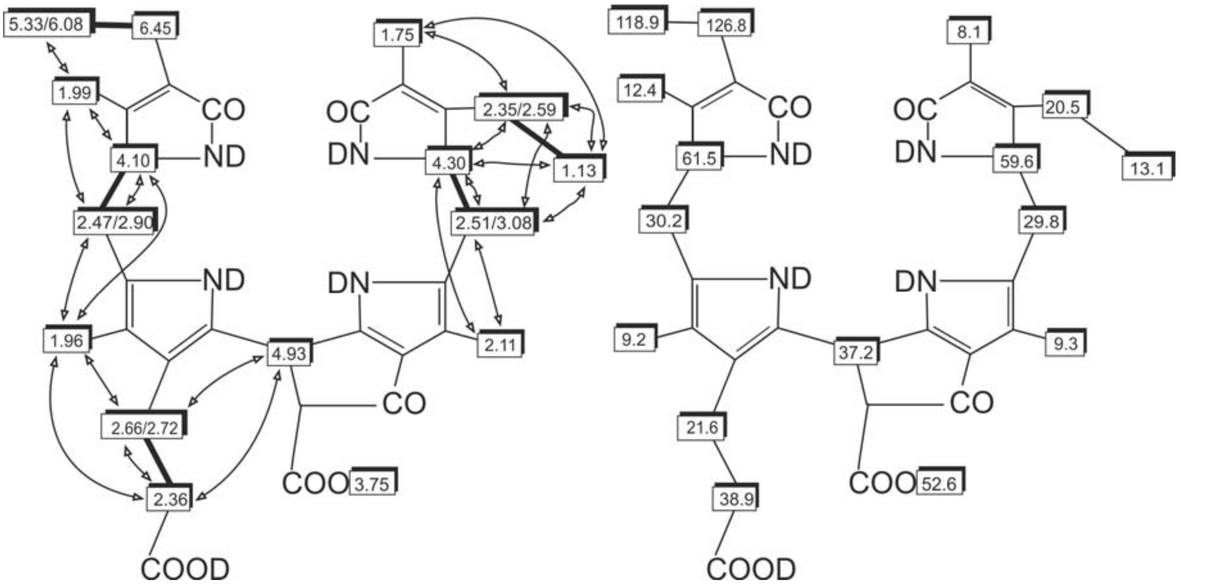

Figure S6. Graphical structural analysis of *At-mes16*-DNCC-47 (**6**). Left. ^{1}H-chemical shift assignments from ^{1}H,^{1}H-ROESY correlations (shown by arrows) and COSY correlations (indicate by bold bonds). Right. ^{13}C chemical shift assignments from ^{1}H,^{13}C-HSQC correlations are shown by shaded boxes.

<u>Spectral data of *At-mes16*-7HM-*iso*-dFCC (7):</u> $3^1,3^2$-didehydro-7-hydroxymethyl-1,4,5,10,17,18,20,22-(7H,21H,23H)-octahydro-13^2-methoxycarbonyl-4,5-seco-5-nor-4,6-dioxo-phytoporphyrinate (Figure 4). UV/Vis ($c = 1.8{*}10^{-5}$ M): λ_{max} (ε_{rel}) = 358 (1.00), 222 (2.14). CD ($c = 1.8{*}10^{-5}$ M): $\lambda_{min/max}$ [nm] ($\Delta\varepsilon$) = 356 (7.77), 320 (2.54), 304 (4.68), 280 (2.4), 255 (5.50), 214 (-15.5). NMR (see Figure S7): 600 MHz ^{1}H-NMR: δ [ppm] = 1.05 (s, H_3C-7^1), 1.07 (t, J = 7.6 Hz, H_3C-8^2), 1.13 (d, J = 7.3 Hz, H_3C-18^1), 1.63 (m, H_AC-17^1), 1.94 (m, H_BC-17^1), 2.11 (s, H_3C-2^1), 2.16 (s, H_3C-12^1), 2.20 (m, H_2C-17^2), 2.23 (m, H_2C-8^1), 2.42 (m, HC-17), 2.65 (dd, J = 17.8/8.8 Hz, H_AC-20), 2.73 (m, HC-18), 3.05 (dd, J = 17.8/3.6 Hz, H_BC-20), 3.62/3.64 (AB-system, J = 12.3, H_2C-$7^{1'}$), 3.68/3.72 (AB-system, J = 16.8, H_2C-10), 3.74 (s, H_3C-13^5), 4.68 (dd, J= 8.8/ 3.6 Hz, HC-1), 5.37 (dd, J = 11.7/2.2 Hz, H_AC-3^2), 6.18 (dd, J = 17.6/2.3 Hz, H_BC-3^2), 6.50 (dd, J = 17.7/11.6 Hz, HC-3^1); 150 MHz ^{13}C-NMR: δ [ppm]= 8.9 (12^1), 12.4 (2^1), 15.2 (8^2), 17.6 (8^1), 17.7 (7^1), 17.9 (18^1), 23.0 (10), 29.4 (17^1), 33.0 (17^2), 34.6 (20), 48.0 (17), 51.5 (18), 52.7 (13^5), 56.3 (7), 58.5 (1), 65.9 ($7^{1'}$), 113.3 (12), 119.2 (3^2), 123.8 (8), 127.1 (13), 126.9 (3^1), 129.1 (3), 132.8 (9), 135.1 (11), 156.6 (2), 170.5 (13^3), 175.3 (4), 179.4 (17^3), 185.1 (6), 186.5 (19). ESI-MS: m/z (% intensity, type of ion) =685.1 (22, $[M+K]^+$); 669.2 (14, $[M+Na]^+$); 649.1 (18), 648.2 (49), 647.2 (100, $C_{35}H_{43}N_4O_8^+$, $[M+H]^+$); 617.2 (22, $[M-CH_2O+H]^+$); 615.0 (17, $[M-CH_4O+H]^+$); 585.20 (12, $[M-C_2H_6O_2+H]^+$); 494.13 (10, $[M-C_8H_{11}NO_2+H]^+$); 492.13 (10, $[M-C_8H_{13}NO_2+H]^+$).

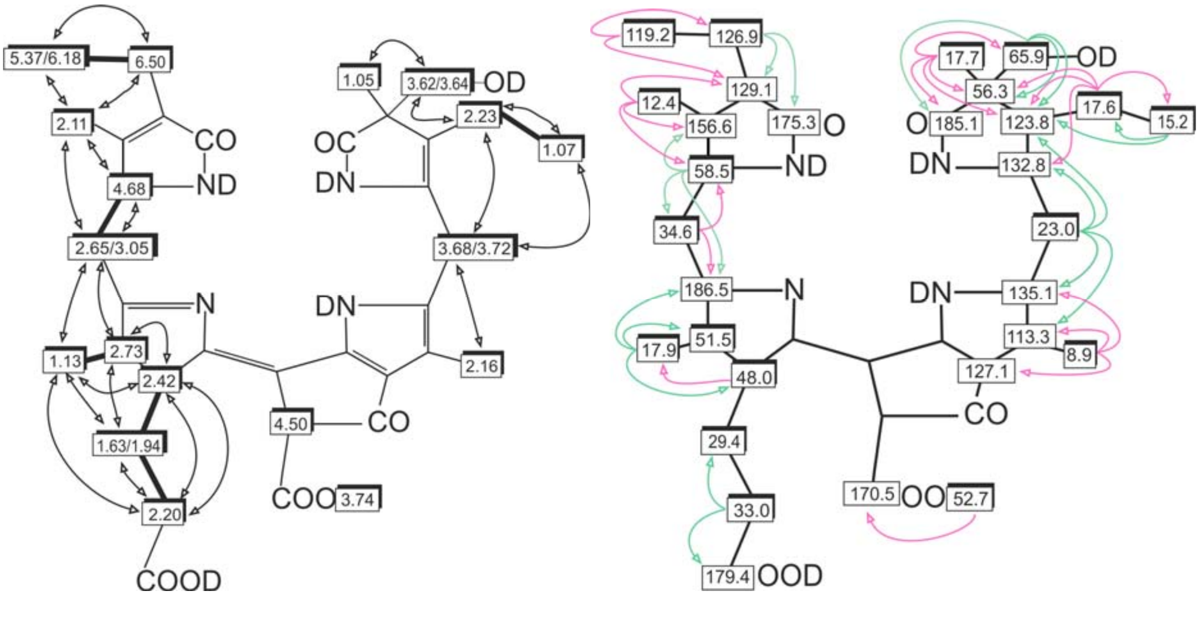

Figure S7. Graphical structural analysis of *At-mes16*-7HM-*iso*-DFCC (**7**). Left. ^{1}H-chemical shift assignments from ^{1}H,^{1}H-ROESY and COSY correlations (arrows or bold bonds, resp.). Right. ^{13}C chemical shift assignments from ^{1}H,^{13}C-HSQC correlations are shown by shaded boxes, and arrows indicate ^{1}H,^{13}C-HMBC long range correlations.

UV- and mass spectral (on-line data) of three minor DNCC-fractions (see Figure 2, main text)

At-mes16-DNCC-37 (**8**): UV/Vis: λ_{max} (ε_{rel}) = 290 sh (0.21), 242 sh (1.00), 212 (1.60). ESI-MS: m/z (% intensity, type of ion) = 671.2 (20, $[M+K]^+$); 655.2 (6, $[M+Na]^+$); 635.2 (12), 634.2 (41), 633.2 (100, $C_{34}H_{41}N_4O_8^+$, $[M+H]^+$); 601.1 (7, $[M-CH_4O+H]^+$); 510.2 (35, [M-C_7H_9NO (ring A)+H]$^+$). *At-mes16*-DNCC-40 (**9**): UV/Vis: λ_{max} (ε_{rel}) = 290 sh (0.21), 242 sh (1.00), 214 (1.59). ESI-MS: m/z (% intensity, type of ion) = 671.2 (28, $[M+K]^+$); 655.2 (11, $[M+Na]^+$); 635.2 (14), 634.2 (40), 633.2 (100, $C_{34}H_{41}N_4O_8^+$, $[M+H]^+$); 601.2(28, $[M-CH_4O+H]^+$); 510.2 (11, [M-C_7H_9NO (ring A)+H]$^+$); 478.1 (6, $[M-C_8H_{13}NO_2+H]^+$). *At-mes16*-DNCC-42 (**10**): UV/Vis: λ_{max} (ε_{rel}) = 290 sh (0.28), 242 sh (1.00), 214 (1.34). ESI-MS: m/z (% intensity, type of ion) = 671.1 (30, $[M+K]^+$); 655.2 (14, $[M+Na]^+$); 635.1 (11), 634.1 (37), 633.1 (100, $C_{34}H_{41}N_4O_8^+$, $[M+H]^+$); 601.0 (23, $[M-CH_4O+H]^+$); 510.0 (12, [M-C_7H_9NO (ring A)+H]$^+$); 478.1 (6, $[M-C_8H_{13}NO_2+H]^+$).

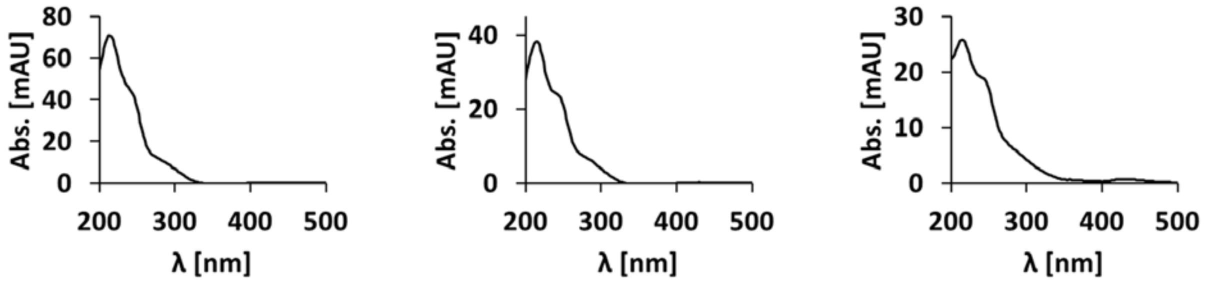

At-mes16-DNCC-37 (8) *At-mes16*-DNCC-40 (9) *At-mes16*-DNCC-42 (10)

Figure S8. Online UV/Vis spectra of three minor DNCC-fractions, which are all isomers of the major catabolite *At*-mes16-DNCC-38 (**3**)

References

[1] Christ, B., Schelbert, S., Aubry, S., Süssenbacher, I., Müller, T., Kräutler, B. and Hörtensteiner, S. (2012) *Plant Physiol.* 158, 628-641.

[2] Gottlieb, H.E., Kotlyar, V. and Nudelman, A. (1997) J. Org. Chem. 62, 7512-7515.

[3] Ernst, R.R., Bodenhausen, G. and Wokaun, A. (1987), *Principles of Nuclear Magnetic Resonance in One & Two Dimensions*, Clarendon Press, Oxford.

[4] Kessler, H., Gehrke, M. and Griesinger, C. (1988) Angew Chem Int Ed 27, 490-536.

[5] Fenn, J.B., Mann, M., Meng, C.K., Wong, S.F. and Whitehouse, C.M. (1989) *Science* 246, 64-71.

[6] Scheer, H., (2006) in: *Chlorophylls and Bacteriochlorophylls* - Biochemistry, Biophysics, Functions and Applications (Grimm, B., Porra, R., Rüdiger, W. and Scheer, H., Eds.), *Adv. Photosynth. Res.* Vol. 25, pp.1-26, Springer, Dordrecht, The Netherlands.
